# Laparoscopic removal of swallowable intragastric balloon and conversion to Roux-en-Y gastric bypass with concurrent hiatoplasty: a case report

**DOI:** 10.1093/jscr/rjag564

**Published:** 2026-07-10

**Authors:** Tabata L Tinoco Ortiz, Maximo V Torres Guaicha, Paul M Tovar-Cobos

**Affiliations:** Hospital Metropolitano de Quito, Department of Surgery, Av. Mariana de Jesús s/n & Av. Occidental (Calle B, N31-190), 170521, Quito, Ecuador; Hospital Metropolitano de Quito, Department of Surgery, Av. Mariana de Jesús s/n & Av. Occidental (Calle B, N31-190), 170521, Quito, Ecuador; Universidad San Francisco de Quito, School of Medicine, Av. Diego de Robles & Av. Oswaldo Guayasamín, 170901, Quito, Ecuador

**Keywords:** swallowable intragastric balloon, hiatal hernia, Roux-en-Y gastric bypass, bariatric surgery, obesity

## Abstract

Swallowable intragastric balloon is a minimally invasive bariatric option for obesity, but severe intolerance requiring intervention is uncommon. We report a 41-year-old man with grade I obesity, hypertension, dyslipidemia, and hyperthyroidism who developed severe retrosternal pain, reflux, persistent vomiting, and oral intolerance 5 days after balloon placement. Computed tomography showed appropriate balloon position without migration or perforation but suggested a small hiatal hernia. Due to refractory symptoms, laparoscopic balloon removal was performed. Intraoperative findings revealed a larger hiatal hernia with paraesophageal lipoma than previously identified, prompting simultaneous hiatoplasty and conversion to Roux-en-Y gastric bypass. The patient had progressive postoperative recovery with symptom resolution and improved oral tolerance. This case highlights that severe balloon intolerance may reveal occult hiatal pathology despite inconclusive preoperative studies and demonstrates that single-stage laparoscopic balloon removal, hiatal hernia repair, and definitive bariatric surgery can be a safe, effective strategy in appropriately selected patients.

## Introduction

Swallowable intragastric balloon (SIGB) is a minimally invasive, temporary bariatric intervention for patients with obesity who seek a non-surgical alternative or have failed, refused, or not tolerated conservative or pharmacologic therapy [1]. Although generally considered safe, SIGB may cause adverse effects such as vomiting, gastroesophageal reflux, and abdominal pain, particularly during the first days. Most of these symptoms are self-limited and respond to conservative management [2].

More severe complications, including balloon migration, gastric outlet obstruction, perforation, pancreatitis, or intolerance refractory to medical treatment, are less frequent but may require balloon removal [3]. Endoscopic removal remains as the standard approach [4]. Laparoscopic removal is rarely required and is generally reserved for severe complications requiring surgical repair or when the endoscopic extraction is not feasible [5].

The concurrent need for another intervention, even a non-urgent one, can justify laparoscopic removal as well. We present an example of these rare instances: A case in which balloon removal was combined with incidental discovery of a previously unknown hiatal hernia, followed by simultaneous hernia repair and conversion to Roux-en-Y gastric bypass (RYGB).

## Case report

A 41-year-old male with grade I obesity (body mass index [BMI] 33.96 kg/m^2^), hypertension, dyslipidemia, hyperthyroidism, and chronic gastritis underwent SIGB placement after failed conservative weight-loss treatment. Upper endoscopy performed 2 years earlier had shown mild nodular erythematous gastritis without other abnormalities.

Five days after placement, he developed acute severe retrosternal and epigastric pain associated with gastroesophageal reflux, and ˃10 episodes of vomiting. He remained hospitalized at the institution of balloon placement, receiving intravenous hydration and analgesia; however, symptoms persisted. One day prior to admission, pain intensified to 10/10 prompting discharge and evaluation at a tertiary care center.

On admission, he was hemodynamically stable. Examination showed abdominal distension and epigastric tenderness without peritoneal signs. Laboratory tests were normal. Computed tomography (CT) of the abdomen demonstrated appropriate placement of an intact balloon, without evidence of migration. No signs of perforation or other complications were identified. A small sliding hiatal hernia was reported; however, as will be discussed later, subsequent intraoperative assessment found a larger defect than initially appreciated.

Despite the absence of major complications on imaging, the patient continued to experience severe symptoms. Given their diverse symptoms, treatment options were discussed extensively with the patient. Since he met accepted indications for metabolic-bariatric surgery—class I obesity with associated comorbidities & unsuccessful conservative attempts of weight loss—the possibility of performing definitive bariatric surgery at the time of balloon removal was considered. The patient expressed a preference for this single-stage surgical approach to avoid a second procedure.

**Figure 1:**
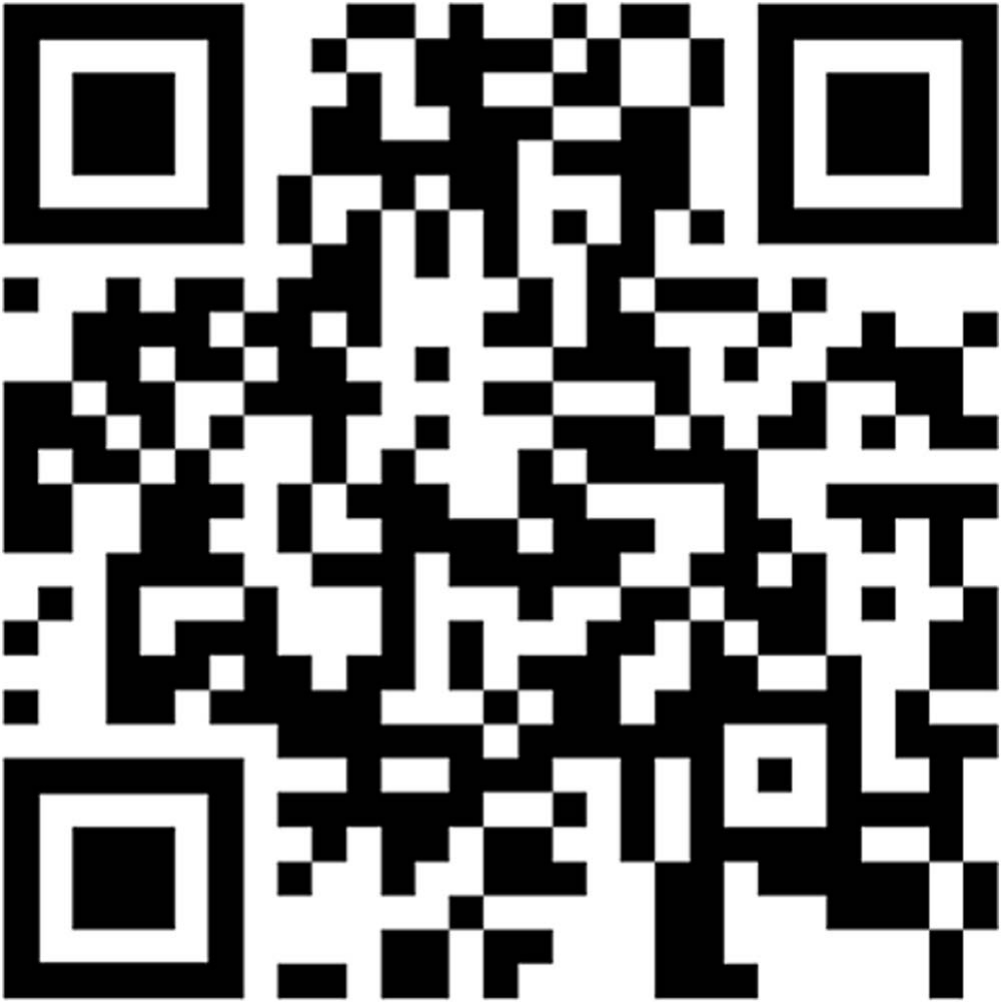
Surgical video.

The patient underwent laparoscopic intragastric balloon removal, with conversion to RYGB and hiatoplasty (Fig. 1).

**Figure 2:**
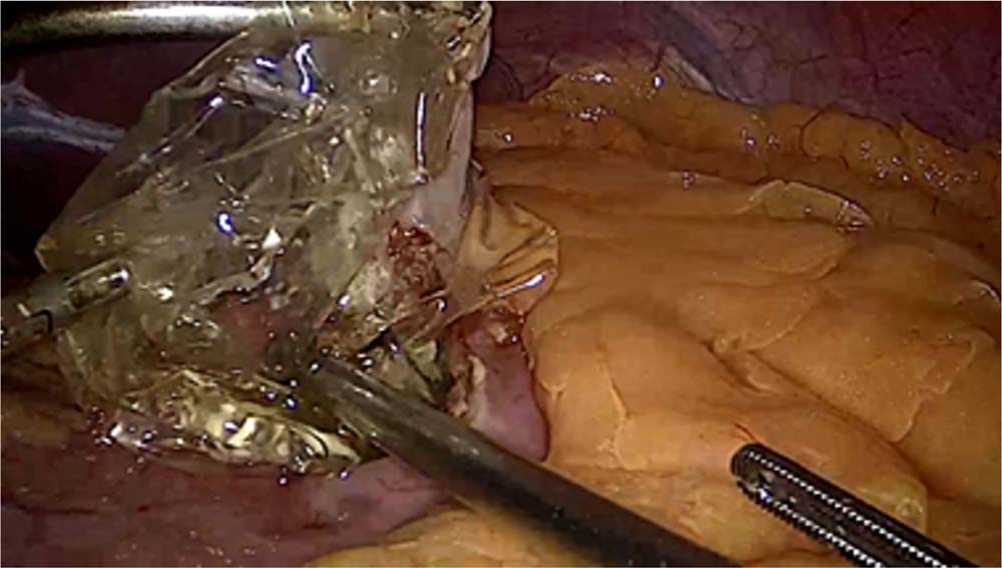
Allurion® Intragastric balloon, removal through laparoscopic gastrostomy.

**Figure 3:**
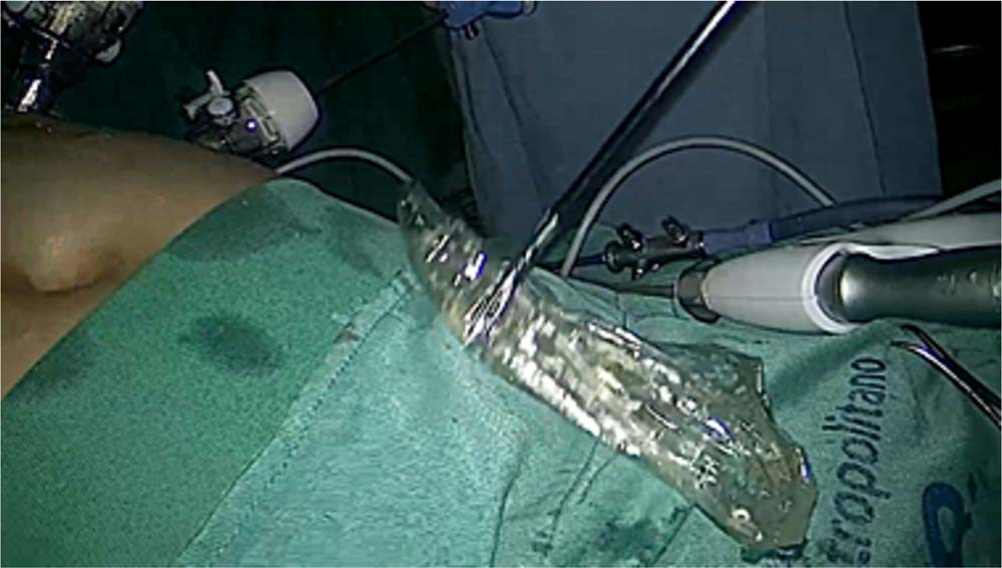
Allurion® Intragastric balloon, integrity confirmed after removal.

During the procedure, the integrity and adequate position of the balloon was confirmed (Figs 2 and 3), and no signs of perforation, bleeding, or other complications were noted.

**Figure 4:**
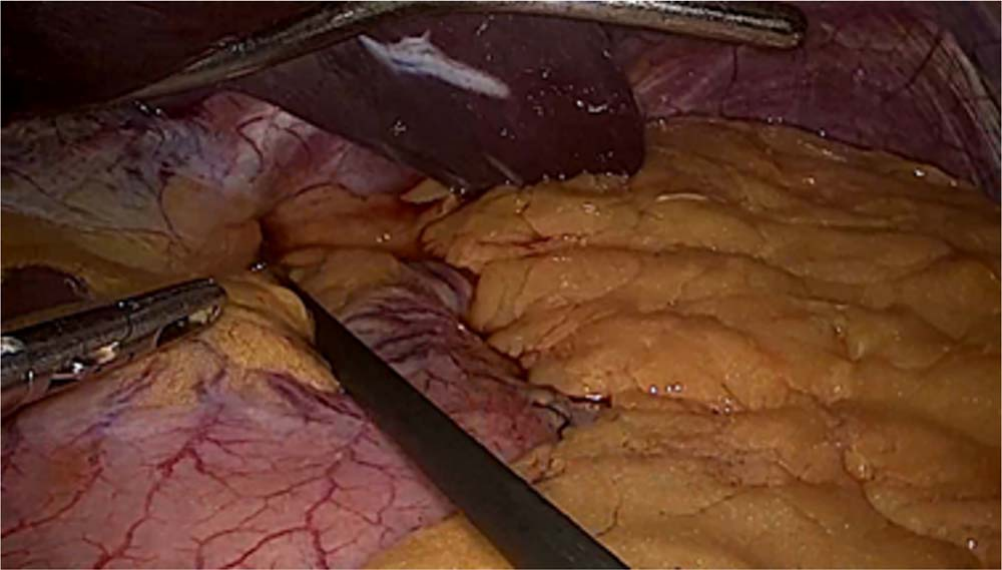
Sliding hiatal hernia.

**Figure 5:**
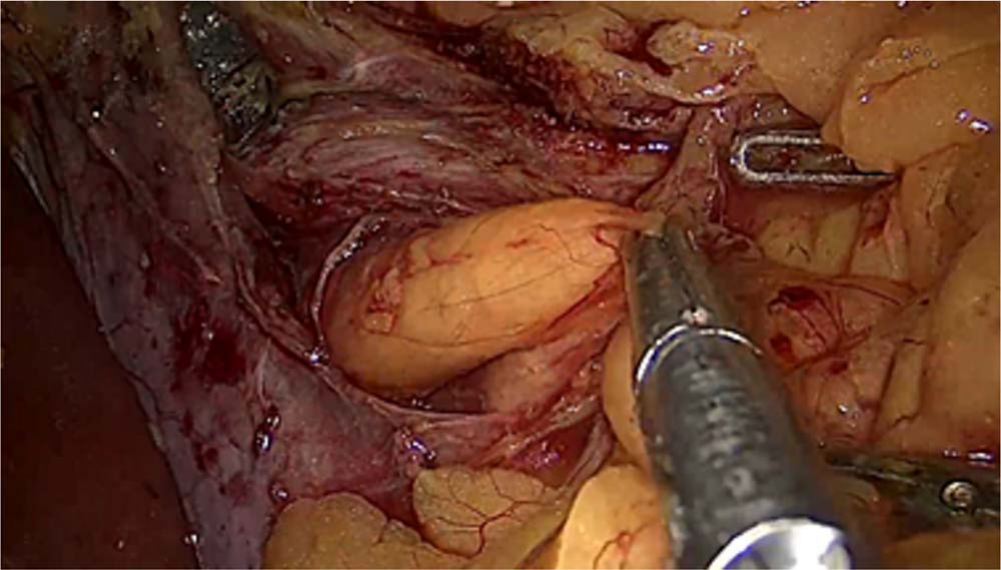
Prehernial lipoma.

The hernia was characterized as a sliding hiatal hernia (Fig. 4) with a large paraoesophageal lipoma measuring ~4 × 3 cm (Fig. 5).

**Figure 6:**
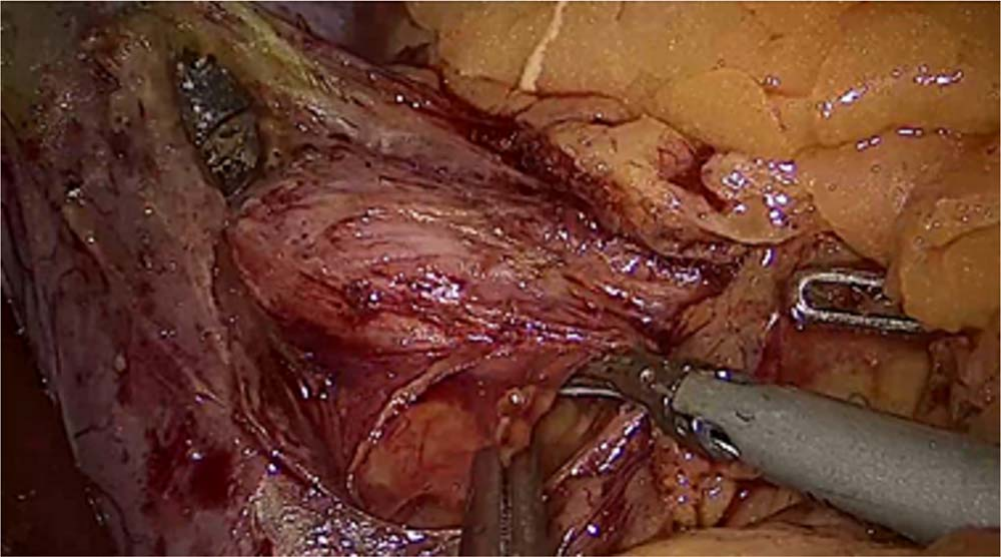
Anterior defect.

**Figure 7:**
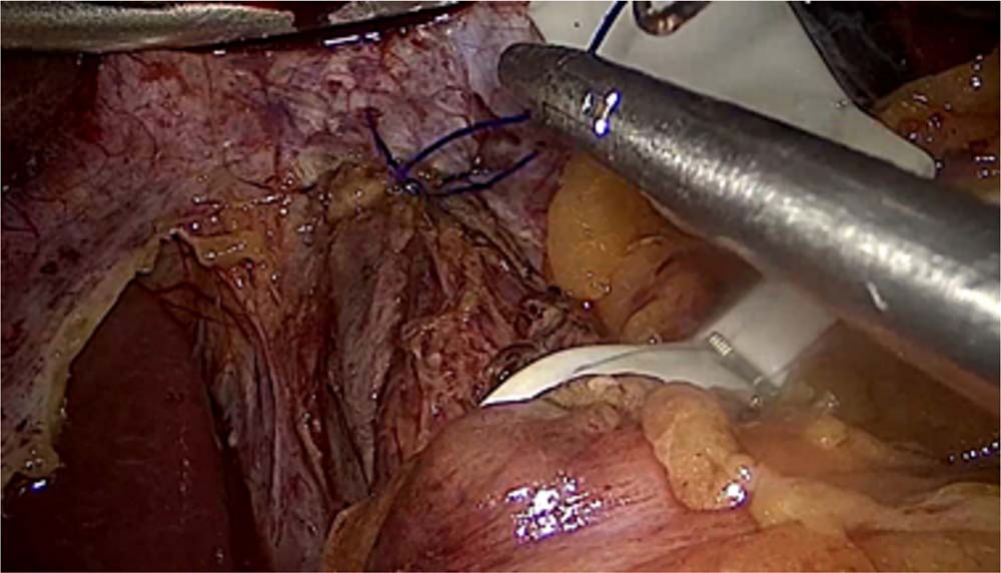
Posterior defect. After dissection of the right and left diaphragmatic pillars.

**Figure 8:**
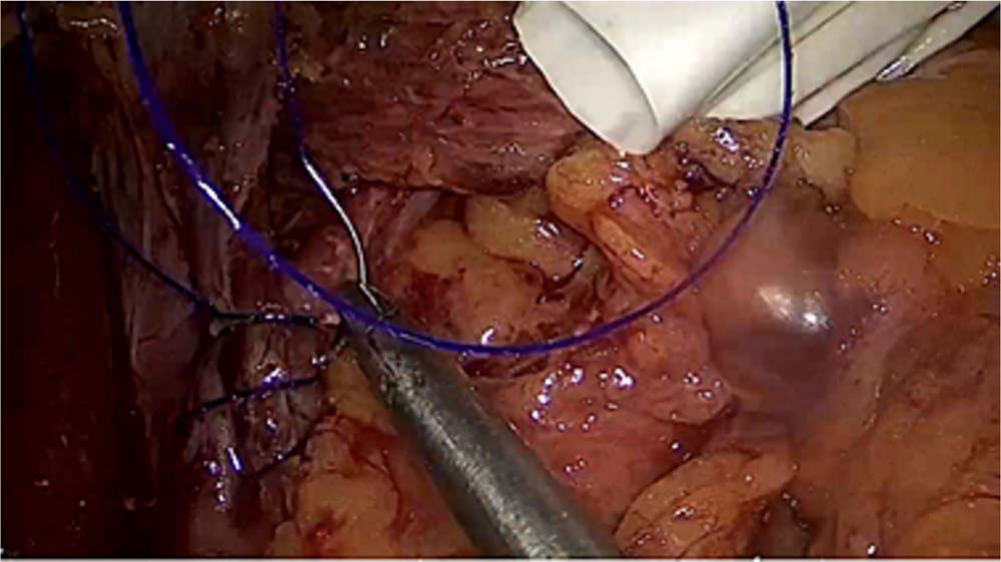
Anterior defect closure using 2/0 non-absorbable barbed suture.

**Figure 9:**
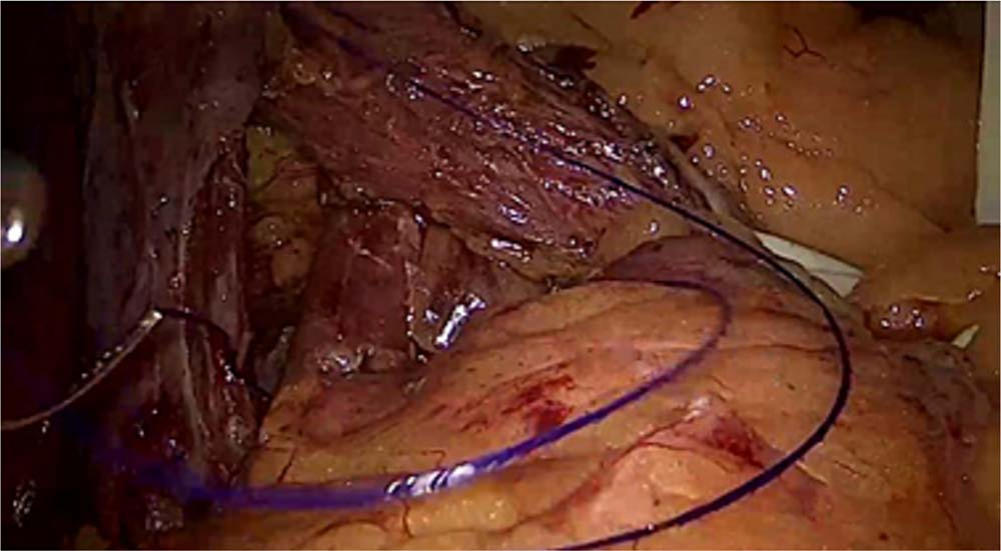
Posterior defect closure using 2/0 non-absorbable barbed suture.

The hernia was repaired successfully without complications. (Figs 6–9).

Postoperatively, the patient improved clinically with resolution of pain and recovery of oral tolerance. However, persistently elevated blood pressure readings were noted despite resumption of his usual medication. Considering his history of hypertension and hyperthyroidism, the Internal Medicine service was consulted.

Evaluation by the Internal Medicine team revealed no clinical or laboratory evidence of infection, sepsis, pancreatitis, perforation, renal impairment, or exacerbation of hyperthyroidism. As no secondary cause requiring urgent intervention was identified. The consulted team considered that the patient’s pre-existing primary hypertension might not have been adequately controlled or that a white-coat effect could contributed to the recordings. As neither possibility required immediate intervention and the patient remained clinically stable, further assessment of blood pressure control was deferred to the outpatient setting. The patient was ultimately discharged with recommendations for follow-up with Bariatric Surgery, Internal Medicine, nutritional counseling, and management of metabolic comorbidities. At 1-month follow-up, the patient tolerated a soft diet without reflux or vomiting, blood pressure normalized, and weight loss progression was satisfactory, reducing the BMI to 31.16 kg/m^2^.

## Discussion

This case illustrates an unusual presentation of SIGB intolerance characterized by retrosternal pain, reflux, and inability to tolerate oral intake shortly after placement. The discrepancy between preoperative evaluation and intraoperative findings is particularly noteworthy. Prior endoscopy had not demonstrated hiatal hernia, a prerequisite for SIGB placement. During acute evaluation, CT revealed only a small sliding defect and no major complications such as migration, perforation, gastric outlet obstruction, or pancreatitis. However intraoperative evaluation proved that the hiatal defect was substantially larger than expected, ~4 × 3 cm.

Several explanations may account for this discrepancy. Small or intermittent hiatal hernias can be missed during endoscopy, especially when the herniation is dynamic or reduced at the time of examination [6]. A meta-analysis found endoscopy has a diagnosing sensitivity of 72% for hiatal hernias when compared to surgical exploration [7]. Likewise, CT may underestimate defect size because it provides only a static assessment and may not fully characterize proximal gastric migration [8]. Additionally, the balloon itself may have increased intragastric pressure and contributed to the manifestation or enlargement of a previously occult hiatal defect.

The decision to perform RYGB was based on several considerations. First, the patient’s BMI of 33.96 kg/m^2^ fell within the accepted criteria for metabolic-bariatric surgery. According to the 2022 IFSO guidelines, metabolic-bariatric surgery should be considered in class I obesity when related comorbidities are present (like the hypertension and dyslipidemia of the patient) and non-surgical therapies have failed [9]. The patient exhibited a history of unsuccessful non-surgical weight-loss attempts. Furthermore, the latest attempt caused intolerance characterized by severe gastroesophageal reflux symptoms, a RYGB was favored over other bariatric interventions such as sleeve gastrectomy since RYGB has consistently demonstrated superior reflux control [10].

Additionally, as no contraindications to bariatric surgery were identified and concomitant hiatal repair was required, a single-stage procedure was deemed an appropriate strategy to achieve both symptom resolution and definitive treatment while avoiding a second operation and further waiting that was unlikely to substantially alter management.

In conclusion, although laparoscopic removal is rarely necessary this case demonstrates it can be safely combined with upper gastrointestinal procedures such as a hiatal hernia repair and RYGB when clinically indicated. A single stage surgical approach may reduce overall morbidities, avoid repeated interventions and provide both symptomatic and metabolic benefits in appropriately selected patients.

This case also highlights the importance of performing bariatric interventions within multidisciplinary programs involving surgical, medical, nutritional, anesthetic, and psychological expertise to optimize safety and outcomes. Ultimately, the safety and success of bariatric therapies depend not only on the procedure itself but also on the comprehensive system of care in which they are delivered.
